# Motor skill intervention for pre-school children: A scoping review

**DOI:** 10.4102/ajod.v9i0.747

**Published:** 2020-12-10

**Authors:** Janke van der Walt, Nicola A. Plastow, Marianne Unger

**Affiliations:** 1 Department of Occupational Therapy, Faculty of Medicine and Health Sciences, Stellenbosch University, Cape Town, South Africa; 2 Department of Physiotherapy, Faculty of Medicine and Heath Sciences, Stellenbosch University, Cape Town, South Africa

**Keywords:** motor skill difficulties, intervention methods, pre-school children, low socio-economic area, framework, scoping review

## Abstract

**Background:**

There is a high prevalence of motor skill difficulties amongst pre-school children living in low socio-economic areas. Motor skill impairment can affect these children’s school readiness and academic progress, social skills, play and general independence.

**Objectives:**

This scoping review investigates the key elements of existing motor skill interventions for pre-school children.

**Method:**

We gathered information through structured database searches from Cinahl, Eric, PubMed, Cochrane, ProQuest, Psych Net, PEDro and Scopus, using a keyword string. The PRISMA-SCR design was used to identify 45 eligible studies. All included studies investigated a motor skill intervention with well-defined outcome measures for children aged 4–7 years with motor skill difficulties. Studies that exclusively focused on children with neurological conditions such as cerebral palsy, physical disabilities or medical/physical deteriorating conditions were excluded. Information was charted on MS Excel spreadsheets. Fundamental concepts were categorised into common key themes and were converted into a proposed framework.

**Results:**

Fifteen intervention approaches were identified. Treatment is mostly managed by occupational therapists and physiotherapists. Evidence supports individual and group treatment with a child-centred, playful approach in a school or therapeutic setting. Whilst session information varied, there is moderate evidence to suggest that a 15-week programme, with two weekly sessions, may be feasible.

**Conclusion:**

Children with motor skill difficulties need therapeutic intervention. This study identified the key elements of existing therapy intervention methods and converted it into a proposed framework for intervention planning. It is a first step towards addressing motor skill difficulties amongst pre-school children in low socio-economic areas.

## Introduction

Motor skills development refers to the acquisition of gross and fine motor skills. Impairment in areas such as balance, coordination and eye–hand coordination may impact on play (Cairney et al. 2010), peer relationships (Wagner et al. 2012), independence skills (Van der Linde et al. 2015) and academic progress (Cameron et al. 2012). These difficulties persist into primary and secondary school (Harrowell et al. 2018), and therapeutic input is essential as children do not just grow out of these difficulties (Hillier 2007). Early support and intervention could help to prevent children failing and reduce the dropout rates throughout the school years (Wills 2016). Unfortunately, motor skill impairment is an often hidden disability.in developmental disorders such as Developmental Coordination Disorder (DCD), Attention Deficit and Hyperactivity Disorder (ADHD), Autism Spectrum Disorder (ASD) and language disorders. Children with Foetal Alcohol Syndrome (FAS) and Human Immunodeficiency Virus (HIV)/Acquired Immune Deficiency Syndrome (AIDS) also experience motor impairments. These two conditions have a high prevalence in low- and middle-income countries (LMIC) (Garrib et al. 2006; Van Rie, Mupuala & Dow 2008; Olivier, Curfs & Viljoen 2016).

Low socio-economic status increases the risk of motor skill impairment amongst children. A Brazilian study used the Movement Assessment Battery for Children (MABC) and found a prevalence of 33% of DCD/probable DCD amongst socially disadvantaged children (4–10 years) (Valentini, Clark & Whitall 2015). A prevalence study in South Africa’s West Coast indicated a prevalence of 14.5% of motor skill difficulties amongst pre-school children (Van der Walt, Plastow & Unger 2020). Valentini et al.’s study (2015) catagorised children who scored below the 15th percentile on the MABC as having probable DCD or being at risk of DCD, whilst Van der Walt et al. (2020) acknowledged a wider range of possible causes under the umbrella term of motor skill difficulties (scores < 15%). These prevalence figures are high when compared to high-income countries (HIC). A population-based study in the United Kingdom using the DSM-IV criteria indicated DCD prevalence of 1.7% at age 7 (Lingam et al. 2009), whilst a recent DCD overview reports that up to 7% of school-aged children have DCD (Caçola & Lage 2019). Morley et al.’s UK-based study (2015) assessed the motor proficiency of children (4–7 years) using the Bruininks-Oseretsky Test of Motor Proficiency-2. Results indicated that low socio-economic status in HIC also significantly affects the development of movement skills (*p* < 0.001).

The motor skills of children living in poverty can further be affected by nutrition, relationships and play opportunities (Worku et al. 2018; Van der Walt et al. 2020). For example, a cross-sectional descriptive prevalence study, using multi-stage clustering by Van der Walt et al. (2020), found that pre-school children with limited access to a playground scored significantly lower on fine motor skill subtests of the MABC-2 than peers who had access to a playground (*p* = 0.009). Scores on balance subtests were also lower. However, scores for ball skills were on par or in some individuals better than their peers who did have playground access (*p* = 0.36). We believe that this is most likely because balls are readily available in these settings, despite poverty.

Literature on motor skill interventions mainly focuses on treatment approaches and programmes used to address difficulties associated with DCD (Camden et al. 2014; Case-Smith, Frolek Clark & Schlabach 2013; Hillier 2007; Mandich et al. 2001; Smits-Engelsman et al. 2018). Other studies focus on Fundamental Movement Skills interventions, which are interventions geared at developing the foundation skills needed for sport participation in school and in later years (Jones et al. 2011; Pope et al. 2011). A systematic review (Veldman, Jones & Okely 2016) investigated the efficacy of gross motor skill interventions in early childhood settings, but excluded studies that included children with health problems or with certain diagnoses, for example, autism, where motor skill difficulties may be co-morbid. The studies highlighted the importance of therapist, teacher and parent involvement as well as methodological sound interventions, whilst also emphasising the lack of quantity and quality interventions aimed at addressing motor skill difficulties. A recent systematic review by Eddy et al. (2019) focussed specifically on the effectiveness of school-based interventions for children aged 3–12 years. The study concluded that, although school-based interventions overall had positive outcomes, the level of benefit depended on the type of intervention. The authors recommend further research to determine dosage and intensity of interventions, and comparison between targeted and universal interventions. Nevertheless, this systematic review only included case-control and randomised studies published between 2012 and 2017.

This scoping review investigates the key characteristics and features of motor skill interventions for pre-school children conducted in any setting, up to May 2019, to inform a best practice model that can be adapted for specific communities. Because of the wide range of diagnoses, types of interventions, disciplines treating motor skill impairment and intervention settings, a scoping review was the preferred methodology (McKinstry et al. 2014; Pham et al. 2014). This review may inform future studies focused on specific interventions or intervention characteristics.

## Method

The aim of this scoping review was to identify the key features of interventions for improving motor proficiency in pre-school children. The study followed the PRISMA-SCR guidelines (Tricco et al. 2018) and the six stages of planning a scoping review as described by Levac et al. (2010).

The following research questions were considered: What interventions exist aimed at improving motor skills in pre-school children? How are these interventions provided in terms of frequency, duration, method, intervention provider and treatment setting? What is the level of evidence for these interventions? and What are the recommendations for implementation of these interventions?

Relevant studies were identified by searching through the following database accessible through Stellenbosch University’s library – Cinahl, Eric, PubMed, Cochrane, ProQuest, Psych Net, PEDro and Scopus – using the keywords motor skills, motor impairment, gross motor skills, fine motor skills, treatment, intervention and children. Filters were applied for database searches – an example of a database search is available (Online Appendix 1). Records were included when available in English or translated to English. Any outcome-based method of intervention aimed at improving motor skills in children between 4 and 7 years of age was included. These included randomised controlled trials (RCTs), case-controlled studies and quasi-experimental studies that aimed to determine the effect of an intervention to improve motor skills and using standardised outcomes to measure effect. Study participants had to present with a motor skill delay or at least a risk of motor skill delay at the onset of a study. Studies investigating only typically developing children and studies that exclusively focused on a neurological condition, physical disability or physical/medical deteriorating condition were excluded. Literature reviews, systematic reviews and meta-analyses were also considered. Grey literature was considered; however, none of these studies adhered to the inclusion criteria. Additional articles/studies were also found using snowballing and pearling (Hadfield 2019) by searching through the references of included studies and following up on alerts from database. Search results were saved and organised in the reference manager software database of Mendeley (Elsevier 2020). The latest database search was completed in April 2019.

**FIGURE 1:**
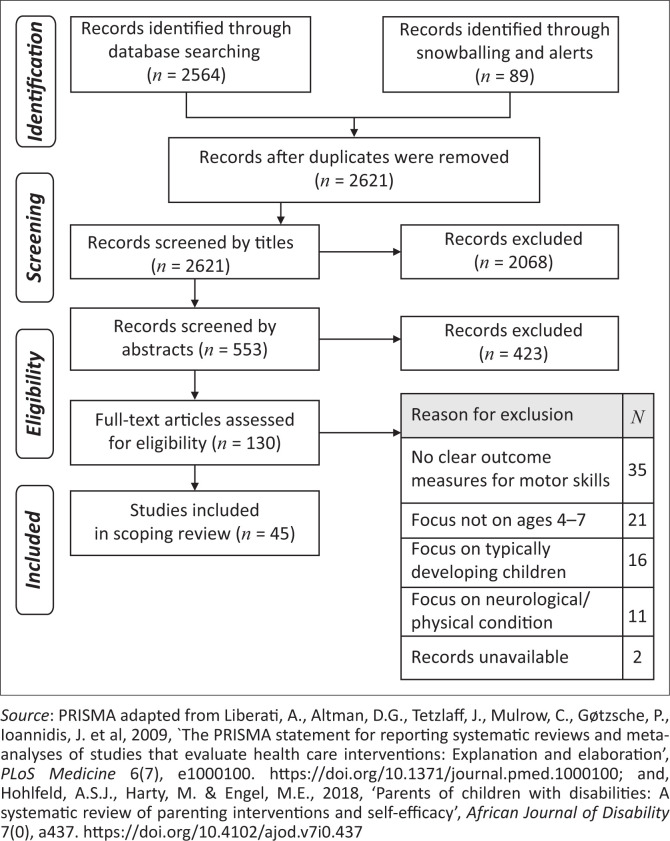
Flow diagram of search strategy.

Records were screened by title and abstract first and then by full articles. The main screening process was carried out by the primary researcher. The records were sent to two co-researchers to review where there was any uncertainty and were included in the study when agreed by both as suitable. Refer to Figure 1 for a description of the search strategy (Liberati et al. 2009).

The researchers developed an MS Excel custom spreadsheet for data capturing. The primary researcher piloted the spreadsheet, which was then reviewed by two secondary reviewers. The final spreadsheet contained 18 main headings. Included studies were classified according to the National Health and Medical Research Council (NHMRC) hierarchy of evidence (Merlin, Weston & Tooher 2009), which grades studies from level I (highest level) to IV (lowest level) – see Online Appendix 2. Critical appraisal of included studies was not done as these are not typically completed in scoping reviews (Arksey & O’Malley 2005; Pham et al. 2014).

The nature of each study was analysed by type (research method) and theme (main idea of the study). Numeric coding was used to assign each study to a category by deductive analysis. Demographic information was charted according to numeric codes developed as records were analysed. The same process was used to plot data relating to interventions (venue, facilitator, structure and equipment required). For diagnoses and treatment approaches, inductive reasoning was applied to list all possible options to incorporate the possibility for several approaches/diagnoses in a study. This was a fluid and progressive process until all records were analysed. Nominal data were input directly for age, group size and session information. Data were analysed by calculating either the total, percentage, mean, median or range according to data sets. Programme duration and session information (quantity, duration and frequency) were calculated across approaches by mean, standard deviation, median and range as can be seen in Online Appendix 2. Additional important information was summarised for each study, categorised and coded accordingly to create a summary of evidence-based recommendations (Box 1).

BOX 1 Evidence-based recommendations for motor skill interventions.

1. **Therapeutic input to improve motor skills**
  - Children with motor skill difficulties need specific therapeutic input to make progress and to avoid regression. They will not grow out of the problem. (Bardid et al. 2013; Goodway, Crowe & Ward 2003; Rintala et al. 1998).
  - Current known interventions used to improve motor skills are overall positive with no one intervention being significantly superior to another. (Case-Smith 2000; Case-Smith et al. 2013; Hillier 2007; Logan et al. 2012; Mandich et al. 2001; May-Benson & Koomar 2010).
  - Cognitive approaches deliver overall positive results. (Mandich et al. 2001; Niemeijer et al. 2006; Smits-Engelsman et al. 2013).
  - Treatment methods based on rhythm and timing have proved to be successful (Bardid et al. 2013; Cosper, Lee, Peters & Bishop 2009; Leemrijse et al. 2000).
  - A pure sensory integration (SI) approach is not indicated but SI principles should be included in an eclectic approach (Smits-Engelsman et al. 2013)
  - Children with Autism Spectrum Disorders (ASD) will benefit from a motor skill programme to address motor skill difficulties (Bremer, Balogh & Lloyd 2015). An SI approach specifically benefit children with ASD (Iwanaga et al. 2014; Pfeiffer et al. 2011)
  - Daily doses of methylphenidate may be useful in children with comorbid ADHD and motor skill difficulties (Bart et al. 2010; Smits-Engelsman et al. 2013).
  - A motor learning approach was shown to be successful. (Bardid et al. 2013; Smits-Engelsman et al. 2013)
  - Structured programmes with specific research-based aims (such as NTT) should be used when developing a programme to address motor skill difficulties rather than unstructured activities such as gaming (Ferguson et al. 2013).
2. **Interpersonal and social approaches**
  - Group therapy and a playful, child-centred approach are indicated as play and peer interaction has a positive effect on motor skill improvement in treatment (Case-Smith 2000; Kirk & Rhodes 2011; Lahav et al. 2008)
  - There is positive evidence for a small therapy group (Smits-Engelsman et al. 2018)
3. **Components of therapeutic input to improve motor skills**
  - Comprehensive occupational therapy intervention results in significant gains in motor and functional skills (Bazyk et al. 2009; Case-Smith 2000; Case-Smith et al. 1998; Case-Smith 1996)
  - Comprehensive and integrated occupational therapy input, using various and/or combined approaches, benefits motor skills as well as literacy skills (Bazyk et al. 2009).
  - Indirect therapy though advice and contributions to a child’s Individual Education Plan (IEP) improves functional skills but does not directly improve fine motor skills (Bayona et al. 2006).
  - A good occupational therapy consultation service (to a school) can have a positive effect similar to a direct therapy service (Dreiling & Bundy 2003).
  - There is positive evidence for a school-based approach (Kirk & Rhodes 2011)

## Results

### Description of included studies

Database searches identified 2564 records with 89 added through snowballing and alerts. Thirty-two duplicates were removed. Title and abstract screening reduced the number for full article review to 130. A final number of 45 articles were included in this scoping review (Figure 1). According to the NHMRC hierarchy of evidence (Merlin et al. 2009), there were no studies included in this scoping review that qualified as a Level 1 study given the absence of systematic reviews of *only* RCTs. Eight studies were graded as Level II with evidence from at least one properly designed RCT. Thirty-four studies were classified as Level III (1–3) studies, using pseudo-RCTs, cohort studies and outcome-based studies with non-randomised allocation of concurrent controls, comparative studies with a historical control or interrupted time series without a parallel control group. A literature review that included only Level III studies was classified at Level III. Three studies were case series, with pre-post or post testing, and were classified at Level IV. A complete description of studies with assigned quality levels is available in Online Appendix 2.

Only two studies included in this review were conducted in LMIC, namely South Africa (Ferguson et al. 2013) and Iran (Najafabadi et al. 2018). Twenty-four studies were conducted in the United States of America, four studies each in Canada and Israel and three in the Netherlands. The remaining studies were from Japan (2), Belgium (2), Australia (2), and one each from Finland and Sweden.

Studies referred to a specific diagnosis or included more than one diagnosis. The diagnostic group most frequently referred to was DCD (14 studies). In 11 studies, children were described as having problems with motor skills, but not diagnosed, and the label of motor skill difficulties was assigned. Studies including children with ASD (10) and ADHD (2) as well as those with developmental delay (11) or being at risk of developmental delay (6) were also described. Other diagnoses included: Down’s syndrome (2), Sensory Processing Disorder (1), Learning Difficulties (1) and Developmental Language Disorder (1).

### Description of data relating to treatment interventions

Studies often referred to more than one intervention approach. The most common approach described was a visual-perceptual motor approach investigated in 30 studies. Fifteen studies referred to a sensory integration approach and 13 to task-specific training. An indirect approach through training, advice, contributing to individual education plans and physical education (PE) was investigated in eight studies and a cognitive-motor approach in another seven studies. Mastery and neuro-motor task training were investigated in four studies. The remaining studies included virtual gaming (3), direct instruction (2), approaches focusing on rhythm and timing (2), pharmaceutical intervention (2), equestrian therapy (1) and body function-orientated input (1). Approaches are described in Online Appendix 2.

All included studies reported to have had a positive influence on motor skills through means of various study designs. Sixteen of the studies explored the effect of a specific approach or programme on the motor skills of children. Eight studies investigated the effectiveness of services or programmes and focussed mainly on positive contributing factors. Five studies investigated the effect of instructional and motivational aspects when implementing a programme. Studies often reported on one approach to be more effective than another according to intervention implementation models or structural elements (6). One study investigated the effect of gender on motor skill intervention. One meta-analysis, three systematic reviews, two combined systematic reviews and meta-analyses and two other comprehensive reviews compared studies for a variety of reasons and described both positive and negative outcomes.

Intervention parameters such as timing and frequency of inputs according to each approach are described in Online Appendix 2. The total number of sessions over all named approaches varied significantly and ranged from 3 to 130 sessions with a mean of 24 and SD of 17 (24 ± 17). The duration of the intervention also varied and ranged from 3 to 40 weeks (15 ± 6). The number of sessions per week ranged from one to five sessions per week (2 ± 1). The session duration varied from 10 to 240 min per session (46 ± 17).

The main facilitators of treatment in these studies were occupational therapists (OTs) (16), followed by physiotherapists (PT) (5) or combined OT and PT input (4). In four studies OTs/PTs and teachers were co-facilitators. Kinesiologists facilitated the treatment in three studies, educators (PE and class teachers) in seven and specialised therapists (e.g. equestrian or hippotherapy) in four. Three studies did not mention who facilitated the sessions.

Intervention programmes were typically carried out in the school environment (17 studies), therapeutic setting (13) or at both (4). Three studies mentioned home programmes as part of an intervention, whilst eight studies did not mention therapy venues. There was poor description of activities used in the interventions and authors were contacted through email to provide more detail (Bazyk 2017). Refer to Online Appendix 2 for detail of activities where these were reported.

**FIGURE 2:**
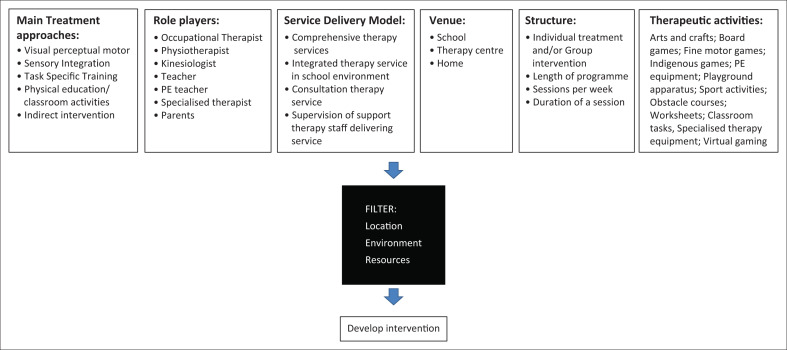
A proposed framework of components to consider for motor skill interventions planning for pre-school children.

The data as described above was summarised in a proposed framework focussing on common features of motor skill interventions for pre-school children (Figure 2). The framework enables one to find and plan motor skill intervention for a specific service or area by ‘filtering’ location, environment and resources. Location refers to the geographical area (e.g. rural vs urban), environment to the conditions under which intervention is planned (educational vs therapeutic, diagnoses or identified difficulties, time, age group, etc.) and resources to funding, equipment and staff available. Important additional information was categorised into the three areas of therapeutic input, interpersonal/social approaches and components of therapy input (Box 1).

## Discussion

To address the hidden disability of motor skills impairment that is experienced by young children with a range of other health conditions, we need to have a framework to inform the choice of approaches that will work best within their own context. This is particularly important when resources, including professional expertise, are limited. The proposed framework, as seen in Figure 2, gives guidance on service and programme development in different contexts, whilst promoting evidence-based practice. We recommend that when developing programmes to address motor skill impairment in young children, professionals consider the treatment approach(es) to be used, the key role players available, how and where services will be delivered, the structure of the intervention programme and the contextually relevant activities that will be used during intervention.

Approaches used in the studies identified in this review varied, but results suggested some positive outcomes in all the studies. This correlates with three systematic reviews (Eddy et al. 2019; Hillier 2007; Logan et al. 2012) that indicated that most interventions have positive outcomes. When looking at specific populations however, certain approaches may be more effective than others. Sensory integration is shown to be effective for children with ASD (Iwanaga et al. 2014), whilst medication may benefit children who experience both motor skill difficulties and attention and concentration deficits (Bart, Podoly & Bar-Haim 2010). A systematic review of high-quality RCTs investigating motor skill interventions for school-going children with DCD found that all effective interventions had a task-orientated approach, but also stated that even within the diagnoses of DCD, heterogeneity should be considered (Preston et al. 2017). A playful, child-centred approach may have a positive influence on motor skill therapy outcomes (Case-Smith 2000; Kirk & Rhodes 2011; Lahav, Apter & Ratzon 2008). For a diverse population with motor skill difficulties, the researchers theorised that a more eclectic approach is recommended to accommodate individual needs (Mandich et al. 2001).

Occupational therapists, followed by PTs and kinesiologists, were found to be the most prominent providers of therapeutic input. It seems to remain a specialist area of care involving a range of specific assessment and treatment approaches. Teachers are often involved together with a therapist, dependent on the service delivery model. In this study, four main service delivery models are described, namely comprehensive OT services; integrated OT services in a school environment; OT consultation services to schools and service delivery by school assistants under supervision of PTs. The level of resources regarding therapists, time and funding is important to consider when implementing best practice. In a low socio-economic area, a task shifting approach (World Health Organization 2008) may be indicated, where teachers are trained to facilitate an intervention with guidance and support from therapists. It should be kept in mind that not all role players in the treatment of motor skill difficulties were included in the review as studies are not available or not fitting the inclusion criteria. Other factors that significantly impact on child development should also be considered (Worku et al. 2018). For example, in areas with a high incidence of alcohol and drug use amongst adults, and high unemployment figures, the involvement of social workers may be beneficial. In rural communities, clinic nurses and doctors may also play a valuable role, whilst pediatricians and child psychiatrists play a role where medication is required (Bart et al. 2010).

From this review, it seems that most interventions occurred either in school settings or at therapeutic centers. Programmes may also include home activities (Hillier 2007). Socio-economic factors, accessibility and therapeutic resources were influencing factors.

Therapeutic activities varied from general arts and crafts (Parush & Hahn-Markowitz 1997), games, sport and gross motor apparatus (Pless et al. 2000) to specialised sensory integration equipment (Iwanaga et al. 2014) and virtual gaming (Salem et al. 2012). One should also consider evidence from a study that suggests that a gross motor programme could have the same effect on fine motor skill development than a programme focusing on fine motor tasks (Parush & Hahn-Markowitz 1997). Although more evidence is needed, such a gross motor skill intervention may simplify the process whilst still offering the same benefits. Looking at dosage parameters, evidence suggests that an intervention programme of 45 min twice a week for 3 to 4 months may be effective.

The country where the studies were conducted should also be considered. Conditions such as DCD, ADHD and ASD are, for example, clearly defined, and diagnostic pathways and treatment regimens are well mapped out within the unique health and education systems in countries such as the United States (CDC 2020) and the United Kingdom (NICE 2020). Diagnostic and intervention pathways in LMIC are less clearly defined and very little statistical information is available regarding developmental diagnostic groups. For example, no prevalence statistics are available regarding DCD or ASD in South Africa (Lamb 2017). It is therefore difficult to focus an intervention programme to a specific diagnostic group when many children with DCD and ASD remain undiagnosed, and many others may have comorbidities such as HIV and FAS affecting motor skills development (Olivier et al. 2016; Smith et al. 2002). In LMIC countries, the term ‘motor skill difficulties’ is also likely to include a wide range of difficulties that may differ from those reported in HICs and thus the outcome of studies from HICs should be interpreted with caution.

The lack of Levels I and II studies (refer to Online Appendix 2) suggests a lack of strong evidence. More RCTs and/or systematic reviews of RCTs, concerning treatment interventions aimed at improving motor skills for pre-school aged children, are recommended to enable more informed decisions regarding best practice interventions for various settings.

## Limitations

Research to date concerning motor skill performance in pre-school children and the effectiveness of treatment methods stems predominantly from HIC. As only English published data were included from limited database in this review, unknown valuable data concerning interventions from developing countries not formally or yet published may exist. There is also little known about the effect of multidisciplinary early intervention collaboration. Although the occupational therapy process seems to be crossing borders with physiotherapy and education, there is even less documented data about other supportive role players such as dieticians, speech and language therapists and psychology services.

## Conclusion

This study identified key concepts that may be associated with successful interventions for improving motor skills in pre-school children. The key concepts were used to assist in developing a proposed framework for intervention design and implementation in a variety of settings. This review and framework may be useful to guide the development of new intervention strategies specific to the needs of a community. The review highlights the need for further research within LMIC and also with regard to other role players as part of the multidisciplinary team.
